# Neural Image Analysis and Electron Microscopy to Detect and Describe Selected Quality Factors of Fruit and Vegetable Spray-Dried Powders—Case Study: Chokeberry Powder

**DOI:** 10.3390/s19204413

**Published:** 2019-10-12

**Authors:** Krzysztof Przybył, Jolanta Gawałek, Krzysztof Koszela, Jacek Przybył, Magdalena Rudzińska, Łukasz Gierz, Ewa Domian

**Affiliations:** 1 Food Sciences and Nutrition, Institute of Food Technology of Plant Origin, Poznan University of Life Sciences, Wojska Polskiego 31, 60-624 Poznan, Poland; kprzybyl@up.poznan.pl (K.P.); jolanta.gawalek@up.poznan.pl (J.G.); magdar@up.poznan.pl (M.R.); 2 Institute of Biosystems Engineering, Poznan University of Life Sciences, Wojska Polskiego 50, 60-625 Poznan, Poland; jacek.przybyl@up.poznan.pl; 3 Faculty of Transport Engineering, Poznan University of Technology, 60-624 Poznan, Poland; lukasz.gierz@put.poznan.pl; 4 Department of Food Engineering and Process Management, Warsaw University of Life Sciences—SGGW, 02-787 Warsaw, Poland; ewa_domian@sggw.pl

**Keywords:** Artificial Neural Network (ANN), classification, image analysis, chokeberry powder, colors, spray-drying

## Abstract

The study concentrates on researching possibilities of using computer image analysis and neural modeling in order to assess selected quality discriminants of spray-dried chokeberry powder. The aim of the paper is the quality identification of chokeberry powders on account of their highest dying power, the highest bioactivity, as well as technologically satisfying looseness of the powder. The article presents neural models with vision techniques backed up by devices such as digital cameras, as well as an electron microscope. The reduction in size of input variables with PCA has an influence on improving the processes of learning data sets, thus increasing the effectiveness of identifying chokeberry fruit powders included in digital pictures, which is shown in the results of the conducted research. The effectiveness of image recognition is presented by classifying abilities, as well as low Root Mean Square Error (RMSE), for which the best results are achieved with a typology of network type Multi-Layer Perceptron (MLP). The selected networks type MLP are characterized by the highest degree of classification at 0.99 and RMSE at 0.11 at most at the same time.

## 1. Introduction

Chokeberry (*Aronia melanocarpa* L.) is a rich source of valuable nutrients, among other things, vitamins and polyphenols. It is also characterized by a high content of antioxidants, which have a positive influence on improving eyesight as well as lower blood pressure [1,2] and lead to early inhibition of cancerogenic stages [3,4,5]. The basic antioxidants that can be found in chokeberry fruit are anthocyanins [6]. Anthocyanins are pigments, which give chokeberry fruit a characteristic dark color [7]. Color intensity depends on pH; when pH is low, the color is intensely red; when pH rises, the color changes into dark blue [8]. Chokeberry also contains valuable tannins (tanning agents), which are responsible for its sensory characteristics, giving chokeberry fruit a very characteristic tart taste [9]. On account of that product obtained from chokeberry, including chokeberry powders, are widely used in functional and pro-health food as well as in all food industries as natural red and dark-blue dye. The still increasing customer awareness in recent years in terms of healthy nutrition leads to an increase in demand for food of natural origin characterized by pro-health properties. At the same time, a dynamically developing trend of foodstuffs fast to make at any moment and in any place, determine the development of instant food technology [10]. Dried vegetable products, especially fruit products, are ideal semifinished goods used in the production of this type of food. Fruits could be dried as whole fruits or their particles, pastes, and juices. A very disadvantageous phenomenon regarding dried food products is the shrinkage of dried material, so changes in the shape and texture of the finished dried product are related to the breaking of capillaries during the drying process [11]. In the case of drying paste and fruit juices, the most commonly used method is spray-dried method with carrier five such powders on a large scale is not easy due to their stickiness [12].

These phenomena are linked with ahigh content of simple sugars and organic acids, which are characterized by the low temperature of verification (Tg). During the process of spray-drying, powders of different morphology and particle microstructure are formed [10,13]. It results from different conditions of drying such as the way and conditions of spraying liquid (i.e., rotary atomizer and its rotary speed), inlet temperature of drying air (inlet and outlet air temperature), physicochemical parameters of feed to be dried [13]. One of the examples is increasing the inlet air temperature, which causes an increase in the speed of drying, and at the same time, causes lower shrinking of particles [14]. In consequence, powder particles are characterized by bigger size and other microstructure are formed [15]. When the inlet temperature of the spray-dried powder is low, the majority of particles show withered surfaces while increasing the inlet temperature results in a larger number of particles of smooth surfaces [16]. A different situation occurs when rotary atomizer speed, then there is a tendency to form particles characterized by smaller sizes [17]. The microstructure of powder particles, in turn, has an influence on functional parameters, which directly determine the abilities of their application. Flowability is one of the most important features of powders because it affects the powders’ behaviors in various processes, e.g., transport, mixing, and dosage [5]. Parameters which have a substantial influence on powder looseness, among other things, are the particle size distribution, loose bulk density, tapped bulk density, the density of the powder particles, the volume of the interstitial air, moisture [5,18,19,20]. A particularly important parameter to characterize features of fruit powders on account of packaging processes, transport, and storing is loose and tapped bulk density. Fleck compaction in powder (compressibility) influences bulk density. Loose bulk density is a volume of loosely poured powdered material, while tapped bulk density that is shaken down is a volume of powder compressed in a normalized way (empty volumes between particles are eliminated) [21,22,23]. Apart from numerous physical features of fruit powders, bioactive features are also crucial, which results from retaining properties of fresh fruit juices from which they are produced. It has an enormous meaning in the application of those powders, as pro-pro-health additives or as natural dyes used in the food and pharmaceutical industry [5,16,20,24]. In industrial production of such powders, a key issue is getting suitable quality, which is determined by given values of various physical and chemical parameters. Marking all parameters in the laboratory is time-consuming; thus a search for fast techniques of assessing the quality of powders during the production process is being continued at all times [10].

In the face of diversity of neural networks resulting from using numerous architectures, a really important stage is the selection of those, which, in an ambiguous way, are able to solve a given problem that is under research. Classification is understood as finding such a classifier, which allows dividing the set of elements into groups, called classes [25,26,27]. Elements belonging to one group are called objects. There can be differences between them, but not in case of properties, on account of which, they were assigned to a given class. One of the examples of neural networks used most commonly in classification issues is Multi-Layer Perceptron (MLP) [28,29]. It is a unidirectional network using a method of learning with a teacher [30,31], possessing a multi-layer architecture with at least one hidden layer [10]. The only possible communication is the one between neurons in adjacent layers. The activating function for hidden neurons has a non-linear character (sigmoid character) [32].

The aim of the research was to provide assessment and quality identification of chokeberry powders on the grounds of its dyeing capabilities as well as to gain the highest bioactivity (i.e., the highest content of anthocyanin and the highest value of antioxidant potential). In addition to the above, the quality assessment also included measuring the proper level of powders’ looseness (indirectly via microstructure and morphology of particles). In order to achieve this goal, a vision technique with a digital camera was used, using dying power on the outer surface as well as electron microscopy using the shapes of the inner structure (morphology structure) of spray-dried chokeberry juices. The utilitarian goal was to devise a neural model capable of classifying research samples of chokeberry powders, which does not stand out from industrial patterns with defined dyeing and bioactive properties and were as looseness properties in terms of color and structure. The authors verified the research results for the formulated neural models. The assessment error called RMS served as a quality indicator of the formulated models. The formulated neural models were characterized by a high degree of accuracy classification. Due to the above, the research aim of the paper was executed.

## 2. Materials and Methods

### 2.1. Preparation of Spray-Dried Chokeberry Powders

Drying was performed using a semi-industrial spray dryer—Niro Atomiser type FU 11 DA (Denmark)—which allowed to perform a full equivalent of the industrial process. Commercial, concentrated chokeberry juice (SVZ International B.V., Holland) (65 Brix) was used in the study. Maltodextrin, with two degrees of crystallization, was used as a carrier DE: 8 and 22 (Roquette Freres—France). The content of all powders that were received was identical: 30% of fruit and 70% of the carrier in dry powder mass. The process was performed for the following inlet air temperature: 150, 160, 170 °C, and with the following values of the rotary atomizer’s rotational speed: 12,000, 13,000, 14,000 rpm. Table 1 presents the experimental conditions used for the spray-drying of chokeberry juice with the carrier.

### 2.2. Image Preparation

The study concentrates mainly on the comparative analysis of chokeberry powders received in various variants of spray drying in accordance with Table 1. Different parameters of drying simultaneously determine different functional properties of powders, that is why the 6 test trials were called quality classes (AR_1, AR_2, AR_3, AR_4, AR_5, and AR_6). The first step with a digital camera and site for image acquisition [10] required acquiring colored 24-bit images that have 4928 × 3264 resolution of color space models RGB (Red Green Blue). One of the methods of computer image analysis was used, i.e., segmentation, that allowed receiving 3050 objects in the form of image patterns that had 450 × 450 resolution. Table 2 presents the layout of objects occurring in given classes of spray-dried chokeberry juice.

Image histogram was used in the research, which allows characterizing image globally on the basis of encoded numerical data. Parameters determining histogram are measurements and statistics of the image such as average, standard deviation, median, minimum, and maximum. Using the author’s original software “PID system”, recommended parameters for file (with .csv extension) were extracted, creating the so-called training set [10,33]. The software allows distinguishing recommended features from image included in color space model RGB [34], as well as H_S_B_L (Hue Saturation Brightness Lightness) and YCbCr (Y is luma component and CB and CR are the blue-difference and red-difference chroma components, respectively) [35,36]. The selected model of colors was achieved on the basis of the mathematical conversion of the RGB color. Table 3 and Table 4 variable input data present image descriptors determining the aforementioned numerical data, which are encoded in color space models. The procedures of image processing were presented in previous research on strawberry powders [10].

**Figure 1:**
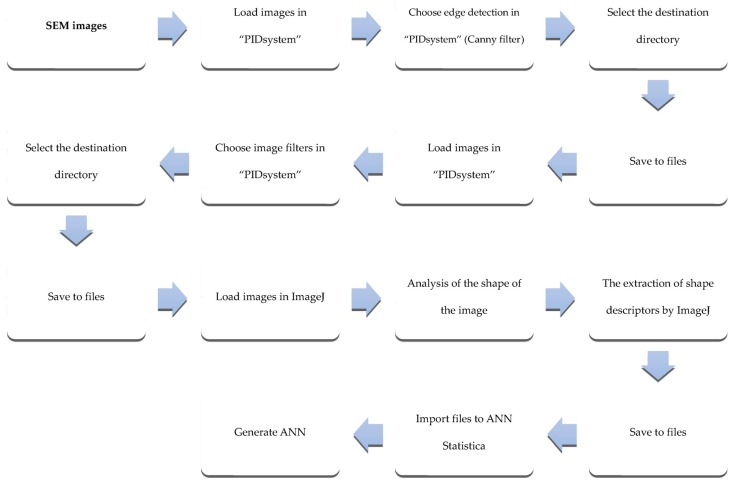
Scheme of the procedure to determine the shape factors to obtain a neural model based on scanning electron microscopy (SEM) images.

**Figure 2:**
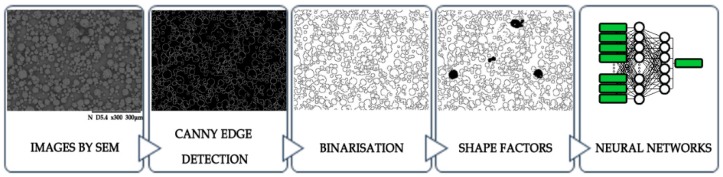
Scheme of shape factors which were extracted of image to the learning process of Neural Networks.

Within the comparison of the efficiency of recognizing chokeberry powders, digital images with the technique of electron microscopy were used. While the research, fragments of fruit powder were indicated, achieving 24-bit images within a range of grey color that has 480 × 390 resolution. On the basis of 12 images, two from each class of chokeberry powders quality, 7406 micro-particles were marked from an image. Micro-particles were achieved with an electron microscope Hitachi TM3000 Tabletop scanning electron microscope (Hitachi High-Technologies Corp., Tokyo, Japan). The study design of acquiring scanning images was achieved on the basis of the previously conducted research on chokeberry powders [5]. The process of processing scanning images consisted of several stages, whose aim was to create image convolution with a Canny filter [33], making image binarization [33] as well as extracting features of shape coefficient with micro-particles occurring on the image (Figure 1 and Figure 2) [33].

### 2.3. Canny Edge Detection

Filter application in image processing is a well-known solution in determining, among other things, edges, pixel weight, noise reduction, removal of granulation, sharpness improvement, changes in recording format, as well as image resolution. The aim is to gain information encoded in the bitmap, as well as determining meaningful image pixels. The Canny method of detection is nothing else but automatic detection of the layout of particle sizes with image analysis based on local adaptive edge detection [37]. The Canny method optimizes three basis criteria: minimizes the number of faulty detections, ensures precise edges localization as well as generates a single answer for each real edge in the image [38].

Edge detection in the image is a popular and effective solution. Among well-known solutions of image convolution, apart from the Canny’s, one can also mention, Roberts, Prewitt, Kirsch, Sobel, Robinson [39,40], Laplace [41], and Laws [10,42]. Convolution filtration of digital images depends on determining derivatives of numerical values of the image with proper selection go color change or gradient change in such a way to achieve adequate sample standard. The primary image is subject to image filtration with the use of masks of 3 × 3, 5 × 5, or 7 × 7 that is applied. In order to apply the Canny filter and to achieve micro-particle edges on image, the original software called “PID system” was used [33].

### 2.4. Binarization

In another step, the image from the Canny filter undergoes binarization obtaining the 1-bit image. Binarization is one of the most important actions of spot image processing [43,44]. It most cases, it precedes image analysis, and it is also very useful in the process of their recognition. Only in case of binary images, it was possible to take the measurements determining the shape coefficient of micro-particles. In order to do this, the Matlab environment with library Hough transform was used [45]. Hough Transform is a very simple method of detecting regular shapes (circles, lines) on the image. This action was tested with ImageJ software [46]. The aim of binarization was a radical reduction of information included in the image as well as extracting shape coordinates of micro-particles.

### 2.5. Image Acquisition

**Figure 3:**
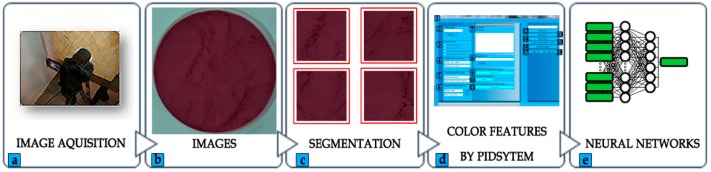
Scheme of color features which were extracted from the image to the learning process of Neural Networks: (**a**)—taking pictures using the research stand; (**b**)—creating of base of digital photos of chokeberry fruit powders; (**c**)—image segmentation; (**d**)—1—image processing module in “PID system”, 2—loading images in the “PID system”, 3—batch image processing selection, 4—determination of image coordinate values (x, y) to be cropped, 5—selection of image format, 6—selection of image pattern to change the name, 7—files list, 8—saving images to the destination directory, 9—extracting color characteristics from images (RGB, YCbCr), 10—approval of the processing process, 11—cleaning the list of objects from the file list window, 12—creating Laws masks, 13—creating Kirch masks, 14—creating Prewitt masks, 15—creating GLCM script to Matlab, 16—creating convolution mask, 17—loading “.csv” file, 18—saving “.csv” file; (**e**)—learning process ANN.

Images of research material samples were obtained using a Nikon D5100 camera with a 16.2-megapixel sensor (CMOS sensor 23.1 × 15.4 mm—DX format), scialytic tent illuminated by visible light (VIS) of warm white color and a color temperature of 6500 Kelvins (K) (Figure 3). In the CMOS matrix, image-setting (exposure to light) and pixel reaction are the same as in the CCD matrix. What differs them is the way of photo-counting and transferring information about luminance that is subject to processing. In the CMOS matrix, each pixel has its own charge—voltage converter, which allows accessing singular point and further transfer information.

Parameters of the research station were calibrated in the following manner: sensitivity of the camera matrix was set at ISO-125, the diaphragm was set at f/8 while exposure time was set at 1/125 sec; length of the prime lens was set to 70 mm, without flash, white balance was set in manual mode After adequately customizing the parameters to each object subject to imaging and preparing the equipment for image acquisition, we compiled a database in the form of digital images of selected types of fruit powders. For the needs of the research, a previously prepared measurement—research site was selected, which was also used when taking digital images for strawberry powders [10] and rhubarb powders.

### 2.6. Principal Component Analysis

One of the methods of factor analysis is the analysis of main components, which, with the use of statistical methods, helps to reduce huge amounts of variables to a few factors, which are not correlated with each other. The PCA analysis was made, whose aim was to reduce training set dimensions [10]. The matrix of correlation of learning sets was also defined, determining the influence of numerical data changeability of image parameters as well as shape compounds [47,48]. 

## 3. Results and Discussion

### 3.1. Classification

**Figure 4:**
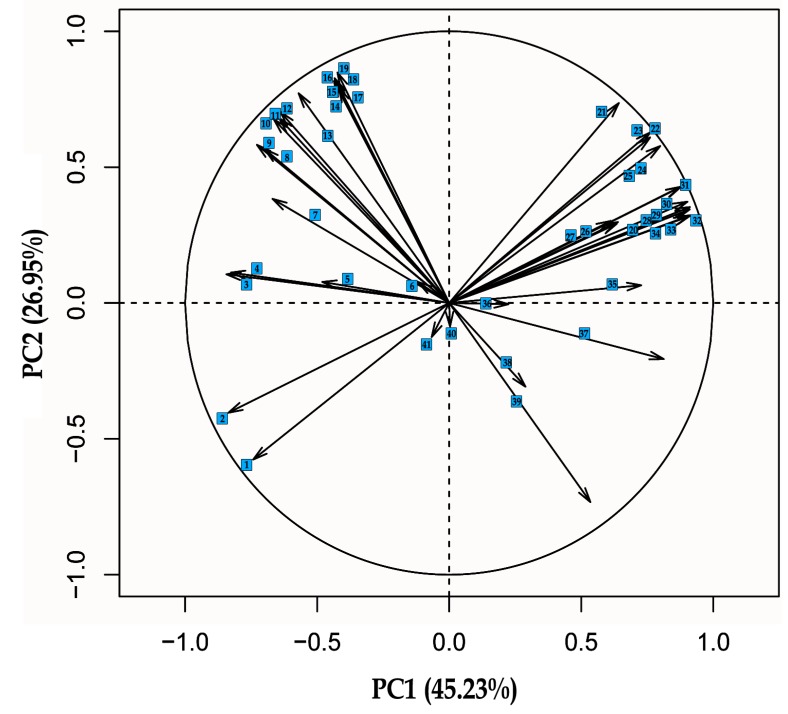
PCA of color features: 1—Saturation Mean, 2—Saturation Median, 3—R Std, 4—Saturation Std, 5—Cb Max, 6—HSV Hue, 7—Cb Std, 8—Brightness Std, 9—Luminance Std, 10—Cr Std, 11—Y Std, 12—B Std, 13—G Std, 14—Luminance Max, 15—R Max, 16—Brightness Max, 17—G Max, 18—B Max, 19—Y Max, 20—Luminance Median, 21—G Mean, 22—B Mean, 23—Y Mean, 24—Luminance Mean, 25—Brightness Mean, 26—Y Min, 27—Luminance Min, 28—B Median, 29—G Median, 30—R Median, 31—R Mean, 32—Y Median, 33—Brightness Median, 34—Brightness Min, 35—Cr Median, 36—Cb Median, 37—Cr Mean, 38—Cb Min, 39—Cr Min, 40—Cb Mean, 41—Cr Max [10,33].

The ANN training process was conducted with network typology type MLP. With a couple of hundred neural networks that were created, the ones characterized by the best classification accuracy were selected, with rather low RMSE. Table 3 shows the results of the training process with sampling of all input variables. The network type MLP was created with 23 input variables, 25 neurons in the hidden layer, and six neurons in the output layer. The MLP 23:23-25-6:1 network was characterized by classification coefficient on the level of 0.92, RMSE = 0.17 and MSE on the level of 0.35. This model presents algorithm BP 50, i.e., the function of learning utilizing algorithm of back-propagating, which in the first stage of learning achieved the lowest level of error in the 50th stage of learning set [49]. Moreover, the conjugate gradient (CG) back-propagation based artificial neural networks for real-time power quality assessment [50]. MLP 23:23-25-6:1 illustrates CG303b, whose aim was to ensure further learning of network during the later stage, as well as to achieve the lowest error causing iteration 303, etc. It is worth paying attention to the fact that the input layer for this model includes 23 image descriptors from model RGB, YCBCR, and H_S_B_L. Dimension reduction of the training set was based on the PCA analysis determining among other things, correlations of input variables (Figure 4) and the analysis of sensitivity for input variables after each process of training was also made for comparison. The MLP 10:10-3-6:1 network is characterized by the worst classification ability, for which the input layer is attributed to the shape coefficient. The shape coefficient was created on the basis of micro-micro-particles distinguished from the image. The influence of applying image filters with the matrix GLCM allowed improving the degree of recognizing the image with colors slightly.

Looking at the level of difficulty in recognizing image for chokeberry fruit powders, the subsequent variants of the selection of input variables were taken into consideration. The models of color spaces were separated in input variables, and trials corresponding output variables were divided regarding conditions that influence the process of spray-dried in accordance with Table 1, i.e., temperature: 150 °C—AR_1, 160 °C—AR_2 and 170 °C—AR_3, rotary atomizer speed:12,000 rpm—AR_2, 13,000 rpm—AR_4 and 14,000 rpm—AR_5 as well as the degree of saccharification of drying carrier (DE): 8—AR_2 and 14—AR_6 (Table 4). The network training process showed the influence of selection of color shape model on recognizing chokeberry powders with the same amount of maltodextrin, not recognizing powders on account of the number of carries being used. It turned out that the dominating color space model in recognizing chokeberry powders is YCbCr. The least meaningful discriminants in the process of training were parameters form models HSB and HSL, which led appropriately to retraining the ANN network. The highest classification abilities of network on the level of 0.99 were achieved for MLP 15:15-25-3:1, MLP 15:15-10-2:1 and MLP 12:12-3-2:1. Inlet air temperature, as well as the degree of saccharification of drying carrier (DE) in the process of chokeberry powders drying, had the fundamental meaning in recognizing image regarding a given factor. As a part of comparison, the results of variants of the drying process were collated. Table 4 shows the results with a digital camera. Table 5 shows the results with a selection of variables determining coefficients of micro-particle shape received with electron microscopy. The aim of the experiment was to compare the influence of temperature on spray-drying process, which concerned tests AR_1, AR_2, AR_3 representing neural model MLP 15:15-25-3:1 in relation to the influence of rotational speed of spray disc (the differences in tests) corresponding to neural model with structure MLP 12:12-2-3:1 and MLP 15:15-5-3:1, and in comparison with the influence of the degree crystallization of drying carrier (DE) (differences in tests AR_2 i AR_6), which matches neural models MLP 12:12-3-2:1and MLP 15:15-10-2:1 in Table 4. A lot of parameters have an influence on the properties of powder that is produced; among other things, the most important parameter in the process of spray drying, apart from the aforementioned factors, is dyeing power. By implication, additional research was done aimed at determining the effectiveness of selecting a model of color space. With PCA analysis, it was possible to reduce variables in a given model of color space (Table 6). In the case of the RGB model validity of variables was determined by 12 parameters from RGB histogram, whereas in the case of YCbCr, these were all parameters from histogram YCbCr. The above information was included in Table 4. The same idea was pointed out in Table 5 on account of particles’ shape, but this time not with the use of a camera but with an electron microscope. By comparing Table 4 and Table 5, one can notice that the technique of computer image analysis was more effective in recognizing sampling material, i.e., chokeberry powders than SEM technique.

The RMS error level is three times worse while identifying image particles than while identifying image color. Nevertheless, the same relationship is visible in recognizing chokeberry powder classes with a given factor of drying process influencing shape and color. The best ability of classifying the output variables is by determining the degree of saccharification, as well as the changes in the temperature during the process of drying chokeberry powders. It confirmed by laboratory research of physicochemical and bioactive parameters. An increase in inlet air temperature in the process of spray drying has a positive influence on changes of micro-particles, i.e., their sudden growth, and this results in increased efficiency of powder as its looseness, but it has negative influence of bioactive properties [5]. In the case of the degree of saccharification of the carrier (DE), substantial statistical changes were noted down in the case of particle shape and powders looseness [5]. The worst ability to classify in case of variation of rotary atomizer speed is also confirmed with a quality assessment conducted in the laboratory. In the case of this given variable form most marked parameters, major statistical quality changes were not noted down [5].

### 3.2. Results of Principal Component Analysis

The PCA analysis was made with a trading set characterized by a color space model with 41 input variables. The set was reduced to the minimum possible set of numerical data of color space models (Figure 4). Table 6 and Table 7 present proprietary values for training sets, on the basis of which main components of PCA, as well as vector effects, were illustrated (see Figure 1 and Figure 2). The aforementioned set with variables was reduced, eliminating input variables, such as Hue, R Max, R Std, Y Min, Y Std, Cb Max, Cb Median, Cb Min, Cb Std, Cr Max, all Brightness, Luminance Median, Min, Std. On the basis of the PCA analysis that was made, ANN was received. The training set basis on shape coordinates with 13 input variables was reduced to 10 input variables. As a result of the reduction, MinFeret, Feret, as well as Area was rejected.

In the second phase, the level of correlation between variables was analyzed. Strong correlation in the training set of color models occurred between variables Y Mean and Y Median, and the correlation coefficient was on the level of 0.951. Equally strongly correlated negative results were received between Y Max and Cr Min and amounted to r^2^ = 0.913. In the RGB model, the strongest correlation is between variables B Max and G Max, on the level of r^2^ = 0.996. Comparing parameters between the models YCbCr and RGB, a strong correlation is between the aforementioned variables, i.e., Y Max and B Max on the level of 0.999. However, a strong negative correlation is between variables G Max and Cr Min on the level of 0.932.

**Figure 5:**
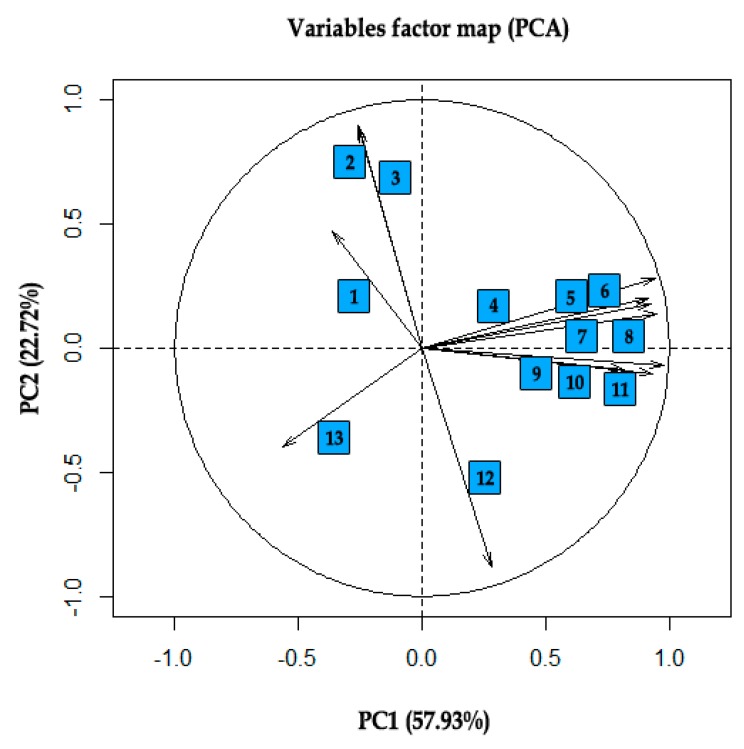
PCA of shape factors: 1—Malinowska factor 2—aspect ratio (AR), 3—Feret factor, 4—Feret diameter X, 5—height, 6—width, 7—W2_circumference 2, 8—perimeter, 9—W1_circumference 1, 10—Feret diameter Y, 11—Area,12—Round, 13—Solidity [10,46].

In case of data variations between parameters in training set of shape coefficient, a strong correlation occurs between Feret and aspect ratio (AR) coefficients on the level of r^2^ = 0.951 and a strong negative correlation of the aforementioned coefficient form Round on the level of r^2^ = 0.815 (Figure 5). 

## 4. Conclusions

Utilization of the two vision techniques with a digital camera and scanning microscope found out that more efficient recognition of quality classes of stay dried chokeberry juice occurs when using color ratio. The best color space model having an influence on the ability to recognize digital images turned out to be the model YCbCr. On the basis of the PCA analysis, it was pointed out that the strongest correlation occurs for the component Y of the model YCbCr. Neural models, in which shape coefficients are included, demonstrated, like in the case of strawberry micro-particles [10], that the efficiency of recognizing micro-particles is dependent on their diameter. On the basis of the PCA analysis, the strongest correlation occurs between Feret coordinates and other input variables determining shape coordinates. Size reduction of input variables in the PCA analysis, improved efficiency of recognizing the image of digital pictures as well as SEM, and at the same time improving the level of trying neural models.

The selection of input variables on account of the selection of factors in the process of drying substantially responds to the improvement of the abilities of the training network. The best-classified parameters in the process of drying were received during recognizing the degree of saccharification (DE) of carrier and different temperatures of drying between trials. It is confirmed by laboratory research of physicochemical and bioactive parameters that were done. An increase in inlet air temperature in the process of spray drying has a positive influence on changes in micro-particles, i.e., their sudden growth, and this results in increased efficiency of fruit powders as well as its looseness; however, it has a negative influence on bioactive properties [5]. In case of the degree of saccharification of the carrier (DE), substantial statistical changes were noted down regarding the size of particles and powder looseness [5]. The worst classification abilities in case variations of rotary atomizer speed are also confirmed by the quality assessment conducted in the laboratory. In the case of this variable for most marked parameters, substantial statistical changes in quality were not detected [5].

The biggest classification abilities were reached by network typology, the Multi-Layer Perceptron. Neural models, which were characterized by the highest quality of degree classification on the level of 0.99, were reached by MLP networks with structure. 15:15-25-3:1, 12:12-3-2:1 and 15:15-10-2:1.

## Figures and Tables

**Table 1:** Experimental conditions used for the spray-drying of chokeberry juice with a carrier.

Powder Code	DE Carrier	Carrier Content (%)	Inlet Air Temperature (°C)	Rotary Atomizer Speed (rpm)
AR_1	8	70	150	12,000
AR_2	8	70	160	12,000
AR_3	8	70	170	12,000
AR_4	8	70	160	13,000
AR_5	8	70	160	14,000
AR_6	22	70	160	12,000

**Table 2:** Layout of frequency of output variables in training set.

Output Variable	AR_1	AR_2	AR_3	AR_4	AR_5	AR_6	AR_ALL
**number of cases regarding color**	496	512	426	484	474	658	3050
**%**	16.26	16.79	13.97	15.87	15.54	21.57	100
**number of cases regarding shape**	978	1266	1150	1370	1464	1178	7406
**%**	13.21	17.09	15.53	18.50	19.77	15.91	100

**Table 3:** Results of training process ANN with a selection of all output variables.

Name	MLP 23:23-25-6:1	MLP 10:10-3-6:1
Input variables	RGB, YCbCR, H_S_B_L	shape factors
Output variables	All AR	All AR
Training error	0.1127	0.3695
Validation error	0.1268	0.3703
Testing error	0.1357	0.3706
Quality of training	0.9443	0.2425
Quality of validation	0.9396	0.2366
Quality of testing	0.9253	0.2408
Training algorithm	BP50, CG303b	BP50, CG150b
Learning cases	3050	7406
RMSE	0.1251	0.3701
MSE	0.3537	0.6084
Accuracy	0.9541	0.2371

**Table 4:** Results of training process ANN with the selection of differences in parameters influencing the drying process (inlet air temperature, rotary atomizer speed, as well as the degree of saccharification of the drying carrier (DE)).

Name	MLP 12:12-2-3:1	MLP 15:15-5-3:1	MLP 15:15-25-3:1	MLP 12:12-3-2:1	MLP 15:15-10-2:1
Input variables	RGB	YCbCR	YCbCr	RGB	YCbCr
Output variables	AR_2, AR_4, AR_5	AR_2, AR_4, AR_5	AR_1, AR_2, AR_3	AR_2, AR_6	AR_2, AR_6
Training error	0.1513	0.1132	0.0798	0.0047	0.0312
Validation error	0.1675	0.1292	0.0814	0.0033	0.0573
Testing error	0.1696	0.2008	0.1665	0.0269	0.0646
Quality of training	0.9538	0.9837	0.9916	0.9999	0.9983
Quality of validation	0.9511	0.9674	0.9944	0.9999	0.9966
Quality of testing	0.9399	0.9344	0.9497	0.9999	0.9967
Training algorithm	BP50, CG300b	BP50, CG45b	BP50, CG48b	BP50, CG229b	BP50, CG76b
Learning cases	1470	1470	1434	1170	1170
RMSE	0.1628	0.1477	0.1092	0.0117	0.0510
MSE	0.4035	0.3843	0.3305	0.1082	0.2259
Accuracy	0.9497	0.9673	0.9940	0.9999	0.9999

**Table 5:** Results of training process ANN shape coordinates.

Name	MLP 10:10-17-3:1	MLP 10:10-47-2:1	MLP 10:10-30-3:1
Input variables	shape factors	shape factors	shape factors
Output variables	AR_1, AR_2, AR_3	AR_2, AR_6	AR_2, AR_4, AR_5
Training error	0.3285	0.2692	0.3277
Validation error	0.3286	0.2763	0.3296
Testing error	0.3293	0.2820	0.3290
Quality of training	0.4255	0.6555	0.4317
Quality of validation	0.4328	0.6285	0.4273
Quality of testing	0.4064	0.6039	0.4322
Training algorithm	BP50, CG67b	BP50, CG382b	BP50, CG66b
Learning cases	3394	2444	4100
RMSE	0.3288	0.2758	0.3288
MSE	0.5734	0.5252	0.5734
Accuracy	0.4328	0.6285	0.4009

**Table 6:** Eigenvalues by color.

	PC 1	PC 2	PC 3	PC 4	PC 5	PC 6	PC 7	PC 8	PC 9	PC 10
Variance	18.544	11.049	3.519	2.441	1.169	0.936	0.781	0.709	0.461	0.315
% of var.	45.229	26.948	8.582	5.953	2.850	2.283	1.905	1.730	1.125	0.767
Cumulative % of var.	45.229	72.177	80.759	86.712	89.563	91.846	93.751	95.481	96.606	97.373
	PC.11	PC 12	PC 13	PC 14	PC 15	PC 16	PC 17	PC 18	PC 19	PC 20
Variance	0.294	0.191	0.134	0.115	0.100	0.050	0.038	0.031	0.028	0.019
% of var.	0.717	0.466	0.327	0.281	0.245	0.121	0.093	0.076	0.067	0.046
Cumulative % of var.	98.090	98.557	98.884	99.165	99.409	99.530	99.623	99.699	99.767	99.813
	*PC.21*	*PC 22*	*PC 23*	*PC 24*	*PC 25*	*PC 26*	*PC 27*	*PC 28*	*PC 29*	*PC 30*
Variance	0.018	0.011	0.009	0.008	0.007	0.005	0.005	0.004	0.003	0.002
% of var.	0.044	0.026	0.023	0.019	0.018	0.012	0.012	0.011	0.007	0.006
Cumulative % of var.	99.856	99.882	99.905	99.924	99.942	99.955	99.966	99.977	99.984	99.990

**Table 7:** Eigenvalues by shape factors.

	PC1	PC2	PC3	PC4	PC5	PC6	PC7	PC8	PC9	PC10
Variance	7.530	2.954	1.233	0.478	0.243	0.202	0.173	0.089	0.043	0.037
% of var.	57.926	22.720	9.488	3.674	1.868	1.551	1.332	0.682	0.334	0.281
Cumulative % of var.	57.926	80.646	90.134	93.808	95.675	97.227	98.558	99.240	99.574	99.856
